# (4*S*)-4-(3,4-Dichloro­phen­yl)-1′-methyl-4′-phenyl-3,4-dihydronaphthalene-2-spiro-3′-pyrrolidine-2′-spiro-1′′-acenaphthyl­ene-1,2′′(2*H*,1′′*H*)-dione

**DOI:** 10.1107/S1600536808013846

**Published:** 2008-05-17

**Authors:** R. Murugan, B. Gunasekaran, S. Sriman Narayanan, V. Manivannan

**Affiliations:** a Department of Analytical Chemistry, University of Madras, Guindy Campus, Chennai 600 025, India; b Department of Physics, AMET University, Kanathur, Chennai, India; c Department of Physics, St. Peter’s Engineering College, Avadi, Chennai, India

## Abstract

In the title compound, C_37_H_27_Cl_2_NO_2_, the 3,4-dichloro­phenyl ring makes a dihedral angle of 46.66 (6)° with the phenyl ring. The mol­ecular structure is stabilized by weak intra­molecular C—H⋯O inter­actions and the crystal structure is stabilized by weak inter­molecular C—H⋯O inter­actions. The C–C–C–C–C five-membered ring is planar, while the C–C–C–C–N five-membered ring adopts a half-chair conformation.

## Related literature

For related lituerature, see: Sarala *et al.* (2006 ▶); Ramesh *et al.* (2007 ▶); Welch *et al.* (1984 ▶). 
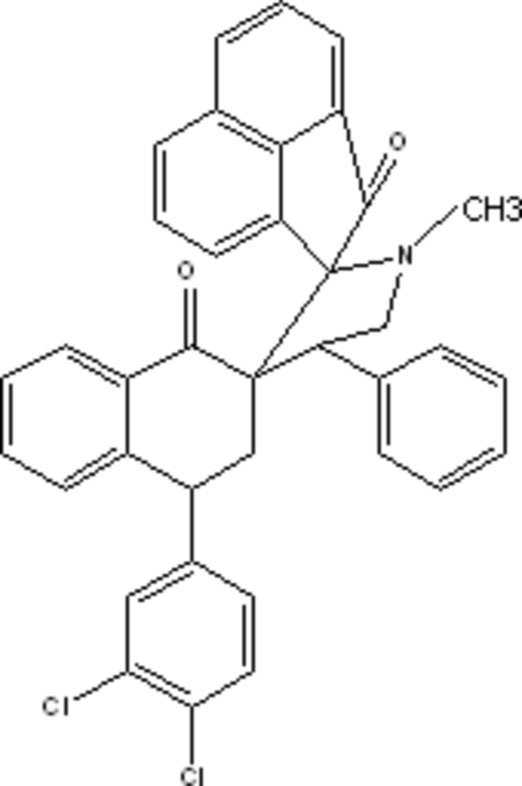


## Experimental

### 

#### Crystal data

C_37_H_27_Cl_2_NO_2_
*M* *_r_* = 588.50
Monoclinic, 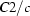

*a* = 39.6142 (12) Å
*b* = 8.3031 (2) Å
*c* = 18.1810 (5) Å
β = 101.135 (3)°
*V* = 5867.5 (3) Å^3^
*Z* = 8
Mo *K*α radiation
μ = 0.26 mm^−1^
*T* = 293 (2) K
0.3 × 0.2 × 0.2 mm

#### Data collection

Bruker APEXII CCD diffractometer
Absorption correction: multi-scan (*SADABS*; Sheldrick, 1996 ▶) *T*_min_ = 0.976, *T*_max_ = 0.979
67467 measured reflections
8160 independent reflections
5776 reflections with *I* > 2σ(*I*)
*R*_int_ = 0.033

#### Refinement

*R*[*F* ^2^ > 2σ(*F* ^2^)] = 0.049
*wR*(*F* ^2^) = 0.152
*S* = 1.06
8160 reflections
380 parameters
H-atom parameters constrained
Δρ_max_ = 0.47 e Å^−3^
Δρ_min_ = −0.32 e Å^−3^

### 

Data collection: *APEX2* (Bruker, 2004 ▶); cell refinement: *APEX2*; data reduction: *SAINT* (Bruker, 2004 ▶); program(s) used to solve structure: *SHELXS97* (Sheldrick, 2008 ▶); program(s) used to refine structure: *SHELXL97* (Sheldrick, 2008 ▶); molecular graphics: *PLATON* (Spek, 2003 ▶); software used to prepare material for publication: *SHELXL97*.

## Supplementary Material

Crystal structure: contains datablocks global, I. DOI: 10.1107/S1600536808013846/bt2710sup1.cif

Structure factors: contains datablocks I. DOI: 10.1107/S1600536808013846/bt2710Isup2.hkl

Additional supplementary materials:  crystallographic information; 3D view; checkCIF report

## Figures and Tables

**Table 1:** Hydrogen-bond geometry (Å, °)

*D*—H⋯*A*	*D*—H	H⋯*A*	*D*⋯*A*	*D*—H⋯*A*
C8—H8*A*⋯O2	0.97	2.49	3.045 (2)	116
C17—H17⋯O1	0.98	2.33	2.794 (2)	108
C32—H32⋯O2^i^	0.93	2.48	3.351 (3)	156

Symmetry code: (i) 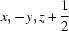.

**Fig. 1.**
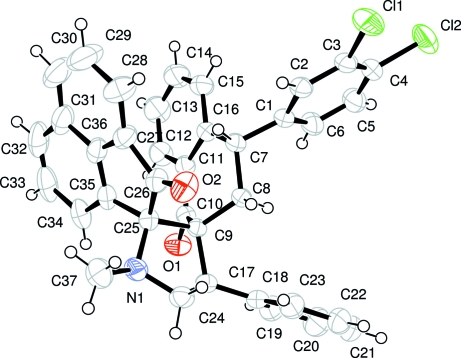
The molecular structure of (I), with atom labels and 50% probability displacement ellipsoids for non-H atoms.

**Fig. 2.**
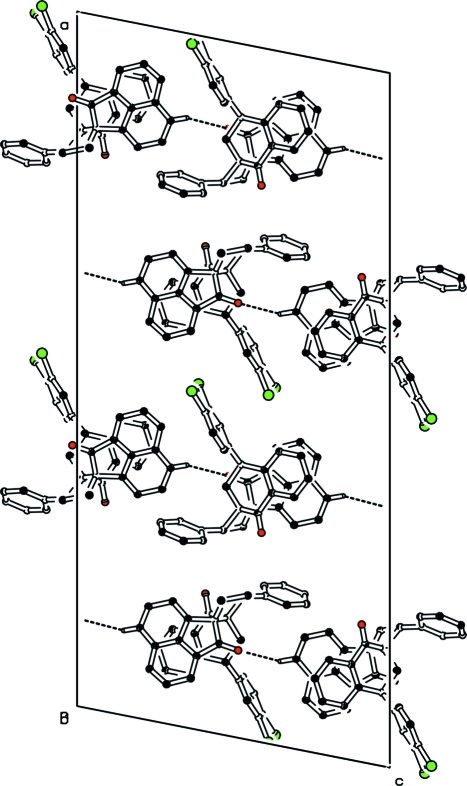
The packing of (I), viewed down the b axis. Hydrogen bonds are shown as dashed lines. H atoms not involved in hydrogen bonding have been omitted.

## supplementary crystallographic information

### Comment

The title compound acts in the central nervous system as a serotonine uptake inhibitor. Another similar compound sertraline hydrochloride is a very effective antidepressant. It selectively blocks serotonine re-uptake and is used for the treatment of depression, as well as dependency and other anxiety-related disorders (Welch *et al.*, 1984).

The geometric parameters in the title compound agree with the reported values of a similar structure (Sarala *et al.*, 2006; Ramesh *et al.*, 2007). The 3,4-dichlorophenyl ring makes a dihedral angle of 46.66 (6) ° with the 4-phenyl ring. The 3,4-dihydro-1(2*H*)-naphthalenone ring adopts half-chair conformation [torsion angles: C(7)—C(16)—C(11)—C(10) -3.61 (3) °, and C(7)—C(8)—C(9)—C(10) -58.20 (17) °].

The molecular structure is stabilized by weak intramolecular C—H···O interactions and the crystal packing is stabilized by weak intermolecular C—H···O interactions.

### Experimental

1.0 mol of (E) -2-benzylidene-4-(3,4-dichlorophenyl)-3,4-dihydronaphthale-1 (2*H*)-one (1.0 g), 1.0 mol of acenaphthaquinoline (0.65 g) and 1.0 mol of sarcosine was refluxed in 10 ml of methanol for about 5.0 hrs. The solvent was removedand the crude solid was recrystallized from ethanol.

### Refinement

H atoms were positioned geometrically and refined using riding model with C—H = 0.93Å and *U*_iso_(H) = 1.2Ueq(C) for aromatic C—H, C—H = 0.98Å and *U*_iso_(H) = 1.2Ueq(C) for CH, C—H = 0.97Å and *U*_iso_(H) = 1.2Ueq(C) for CH_2_, C—H = 0.96Å and *U*_iso_(H) = 1.5Ueq(C) for CH_3_.

### Crystal data

**Table:**

C_37_H_27_Cl_2_NO_2_	*F*_000_ = 2448
*M**_r_* = 588.50	*D*_x_ = 1.332 Mg m^−^^3^
Monoclinic, *C*2/*c*	Mo *K*α radiation λ = 0.71073 Å
*a* = 39.6142 (12) Å	θ = 2.0–25.5º
*b* = 8.3031 (2) Å	µ = 0.26 mm^−^^1^
*c* = 18.1810 (5) Å	*T* = 293 (2) K
β = 101.135 (3)º	Block, pale yellow
*V* = 5867.5 (3) Å^3^	0.3 × 0.2 × 0.2 mm
*Z* = 8	

### Data collection

**Table:**

Bruker APEXII CCD diffractometer	8160 independent reflections
Radiation source: fine-focus sealed tube	5776 reflections with *I* > 2σ(*I*)
Monochromator: graphite	*R*_int_ = 0.033
*T* = 293(2) K	θ_max_ = 29.6º
ω scans	θ_min_ = 1.1º
Absorption correction: multi-scan(SADABS; Sheldrick, 1996)	*h* = −54→54
*T*_min_ = 0.976, *T*_max_ = 0.979	*k* = −11→11
67467 measured reflections	*l* = −25→25

### Refinement

**Table:**

Refinement on *F*^2^	Secondary atom site location: difference Fourier map
Least-squares matrix: full	Hydrogen site location: inferred from neighbouring sites
*R*[*F*^2^ > 2σ(*F*^2^)] = 0.049	H-atom parameters constrained
*wR*(*F*^2^) = 0.152	*w* = 1/[σ^2^(*F*_o_^2^) + (0.0658*P*)^2^ + 5.0424*P*] where *P* = (*F*_o_^2^ + 2*F*_c_^2^)/3
*S* = 1.06	(Δ/σ)_max_ < 0.001
8160 reflections	Δρ_max_ = 0.47 e Å^−^^3^
380 parameters	Δρ_min_ = −0.32 e Å^−^^3^
Primary atom site location: structure-invariant direct methods	Extinction correction: none

### Special details

**Table:**

Geometry. All e.s.d.'s (except the e.s.d. in the dihedral angle between two l.s. planes) are estimated using the full covariance matrix. The cell e.s.d.'s are taken into account individually in the estimation of e.s.d.'s in distances, angles and torsion angles; correlations between e.s.d.'s in cell parameters are only used when they are defined by crystal symmetry. An approximate (isotropic) treatment of cell e.s.d.'s is used for estimating e.s.d.'s involving l.s. planes.
Refinement. Refinement of *F*^2^ against ALL reflections. The weighted *R*-factor *wR* and goodness of fit *S* are based on *F*^2^, conventional *R*-factors *R* are based on *F*, with *F* set to zero for negative *F*^2^. The threshold expression of *F*^2^ > σ(*F*^2^) is used only for calculating *R*-factors(gt) *etc*. and is not relevant to the choice of reflections for refinement. *R*-factors based on *F*^2^ are statistically about twice as large as those based on *F*, and *R*- factors based on ALL data will be even larger.

### Fractional atomic coordinates and isotropic or equivalent isotropic displacement parameters (Å^2^)

**Table:**

	*x*	*y*	*z*	*U*_iso_*/*U*_eq_	
Cl1	−0.002080 (13)	0.34019 (9)	0.38789 (4)	0.06938 (19)	
Cl2	−0.014144 (16)	0.71550 (10)	0.36697 (4)	0.0841 (2)	
O1	−0.19815 (3)	0.41995 (17)	0.58966 (8)	0.0475 (3)	
O2	−0.12583 (4)	−0.06851 (17)	0.48514 (7)	0.0512 (3)	
C9	−0.16950 (4)	0.23492 (18)	0.52002 (8)	0.0299 (3)	
C10	−0.17115 (4)	0.36502 (19)	0.57873 (9)	0.0333 (3)	
C25	−0.16332 (4)	0.06301 (19)	0.56048 (9)	0.0333 (3)	
C17	−0.20508 (4)	0.2125 (2)	0.46665 (9)	0.0346 (3)	
H17	−0.2226	0.2408	0.4959	0.042*	
C1	−0.08342 (4)	0.4158 (2)	0.48236 (9)	0.0357 (3)	
C7	−0.10569 (4)	0.3176 (2)	0.52492 (9)	0.0355 (3)	
H7	−0.0949	0.2116	0.5348	0.043*	
N1	−0.19450 (4)	−0.02695 (18)	0.52975 (9)	0.0425 (3)	
C6	−0.08905 (5)	0.5788 (2)	0.47013 (11)	0.0469 (4)	
H6	−0.1074	0.6281	0.4862	0.056*	
C8	−0.14162 (4)	0.2907 (2)	0.47710 (9)	0.0336 (3)	
H8A	−0.1398	0.2109	0.4391	0.040*	
H8B	−0.1492	0.3905	0.4514	0.040*	
C18	−0.21192 (4)	0.3169 (2)	0.39763 (9)	0.0381 (4)	
C26	−0.12974 (4)	−0.0201 (2)	0.54571 (10)	0.0383 (4)	
C2	−0.05649 (4)	0.3440 (2)	0.45610 (9)	0.0388 (4)	
H2	−0.0526	0.2341	0.4630	0.047*	
C3	−0.03528 (4)	0.4344 (3)	0.41954 (10)	0.0434 (4)	
C11	−0.13780 (4)	0.4193 (2)	0.62382 (9)	0.0365 (3)	
C16	−0.10692 (4)	0.3935 (2)	0.59997 (9)	0.0385 (4)	
C35	−0.15689 (5)	0.0661 (2)	0.64538 (9)	0.0398 (4)	
C36	−0.12327 (5)	0.0156 (2)	0.67380 (10)	0.0442 (4)	
C24	−0.20754 (5)	0.0320 (2)	0.45465 (11)	0.0432 (4)	
H24A	−0.2311	−0.0019	0.4366	0.052*	
H24B	−0.1934	−0.0037	0.4198	0.052*	
C27	−0.10569 (5)	−0.0354 (2)	0.61808 (11)	0.0454 (4)	
C4	−0.04084 (5)	0.5976 (3)	0.40924 (11)	0.0494 (5)	
C23	−0.20506 (5)	0.2687 (3)	0.32921 (11)	0.0517 (5)	
H23	−0.1958	0.1672	0.3246	0.062*	
C5	−0.06793 (5)	0.6693 (3)	0.43455 (12)	0.0529 (5)	
H5	−0.0719	0.7792	0.4275	0.063*	
C15	−0.07627 (5)	0.4379 (3)	0.64733 (11)	0.0572 (5)	
H15	−0.0554	0.4209	0.6322	0.069*	
C19	−0.22596 (5)	0.4685 (3)	0.40187 (13)	0.0539 (5)	
H19	−0.2313	0.5030	0.4469	0.065*	
C12	−0.13751 (6)	0.4898 (3)	0.69397 (11)	0.0521 (5)	
H12	−0.1581	0.5081	0.7098	0.062*	
C31	−0.10852 (7)	0.0127 (3)	0.75047 (12)	0.0620 (6)	
C34	−0.17765 (6)	0.1082 (2)	0.69419 (11)	0.0532 (5)	
H34	−0.2005	0.1372	0.6770	0.064*	
C22	−0.21188 (6)	0.3698 (3)	0.26740 (12)	0.0648 (6)	
H22	−0.2073	0.3351	0.2217	0.078*	
C14	−0.07653 (7)	0.5065 (4)	0.71610 (12)	0.0726 (7)	
H14	−0.0559	0.5357	0.7470	0.087*	
C37	−0.19281 (6)	−0.2006 (2)	0.53935 (14)	0.0607 (6)	
H37A	−0.2155	−0.2451	0.5263	0.091*	
H37B	−0.1832	−0.2258	0.5907	0.091*	
H37C	−0.1786	−0.2457	0.5074	0.091*	
C13	−0.10704 (7)	0.5319 (3)	0.73924 (12)	0.0696 (7)	
H13	−0.1070	0.5779	0.7859	0.083*	
C28	−0.07225 (6)	−0.0877 (3)	0.63682 (15)	0.0686 (6)	
H28	−0.0603	−0.1229	0.6006	0.082*	
C32	−0.13034 (9)	0.0616 (3)	0.79920 (12)	0.0757 (8)	
H32	−0.1220	0.0632	0.8507	0.091*	
C21	−0.22524 (6)	0.5203 (4)	0.27320 (15)	0.0713 (7)	
H21	−0.2296	0.5883	0.2318	0.086*	
C20	−0.23216 (7)	0.5694 (3)	0.34039 (16)	0.0728 (7)	
H20	−0.2411	0.6717	0.3448	0.087*	
C33	−0.16333 (8)	0.1062 (3)	0.77166 (13)	0.0709 (7)	
H33	−0.1771	0.1369	0.8053	0.085*	
C30	−0.07403 (8)	−0.0392 (4)	0.76769 (16)	0.0849 (9)	
H30	−0.0627	−0.0418	0.8175	0.102*	
C29	−0.05687 (8)	−0.0856 (4)	0.71354 (19)	0.0918 (10)	
H29	−0.0340	−0.1173	0.7276	0.110*	

### Atomic displacement parameters (Å^2^)

**Table:**

	*U*^11^	*U*^22^	*U*^33^	*U*^12^	*U*^13^	*U*^23^
Cl1	0.0432 (3)	0.0965 (5)	0.0761 (4)	0.0129 (3)	0.0307 (2)	−0.0009 (3)
Cl2	0.0595 (3)	0.0979 (5)	0.1025 (5)	−0.0085 (3)	0.0344 (3)	0.0410 (4)
O1	0.0376 (6)	0.0514 (8)	0.0557 (8)	0.0082 (6)	0.0145 (5)	−0.0118 (6)
O2	0.0620 (8)	0.0522 (8)	0.0423 (7)	0.0176 (6)	0.0172 (6)	−0.0053 (6)
C9	0.0268 (6)	0.0317 (7)	0.0319 (7)	0.0013 (6)	0.0077 (5)	−0.0011 (6)
C10	0.0334 (7)	0.0320 (8)	0.0362 (8)	0.0021 (6)	0.0108 (6)	−0.0002 (6)
C25	0.0366 (8)	0.0317 (8)	0.0331 (7)	0.0021 (6)	0.0102 (6)	−0.0006 (6)
C17	0.0280 (7)	0.0380 (8)	0.0377 (8)	−0.0003 (6)	0.0058 (6)	−0.0020 (6)
C1	0.0268 (7)	0.0431 (9)	0.0378 (8)	0.0014 (6)	0.0071 (6)	0.0029 (7)
C7	0.0273 (7)	0.0400 (9)	0.0399 (8)	0.0017 (6)	0.0082 (6)	0.0047 (7)
N1	0.0428 (8)	0.0347 (7)	0.0488 (8)	−0.0066 (6)	0.0065 (6)	0.0001 (6)
C6	0.0383 (9)	0.0485 (10)	0.0573 (11)	0.0094 (8)	0.0178 (8)	0.0096 (8)
C8	0.0276 (7)	0.0396 (8)	0.0344 (7)	0.0010 (6)	0.0083 (6)	0.0016 (6)
C18	0.0279 (7)	0.0455 (9)	0.0389 (8)	−0.0036 (6)	0.0013 (6)	0.0006 (7)
C26	0.0429 (9)	0.0324 (8)	0.0406 (9)	0.0075 (7)	0.0103 (7)	0.0020 (7)
C2	0.0313 (7)	0.0447 (9)	0.0410 (9)	0.0037 (7)	0.0082 (6)	0.0004 (7)
C3	0.0280 (7)	0.0638 (12)	0.0392 (9)	0.0030 (7)	0.0089 (6)	0.0001 (8)
C11	0.0386 (8)	0.0358 (8)	0.0352 (8)	−0.0032 (7)	0.0080 (6)	−0.0018 (6)
C16	0.0336 (8)	0.0457 (9)	0.0356 (8)	−0.0050 (7)	0.0055 (6)	0.0045 (7)
C35	0.0531 (10)	0.0342 (8)	0.0339 (8)	−0.0060 (7)	0.0132 (7)	0.0014 (6)
C36	0.0578 (11)	0.0364 (9)	0.0358 (8)	−0.0069 (8)	0.0029 (7)	0.0044 (7)
C24	0.0397 (9)	0.0416 (9)	0.0458 (9)	−0.0068 (7)	0.0020 (7)	−0.0038 (7)
C27	0.0452 (9)	0.0426 (10)	0.0452 (10)	0.0057 (8)	0.0012 (7)	0.0057 (8)
C4	0.0348 (8)	0.0651 (13)	0.0491 (10)	−0.0040 (8)	0.0103 (7)	0.0153 (9)
C23	0.0545 (11)	0.0560 (12)	0.0442 (10)	−0.0072 (9)	0.0084 (8)	−0.0038 (9)
C5	0.0462 (10)	0.0489 (11)	0.0652 (12)	0.0041 (8)	0.0146 (9)	0.0148 (9)
C15	0.0393 (9)	0.0862 (16)	0.0440 (10)	−0.0144 (10)	0.0026 (8)	0.0033 (10)
C19	0.0498 (10)	0.0540 (12)	0.0578 (12)	0.0095 (9)	0.0098 (9)	0.0082 (9)
C12	0.0575 (11)	0.0569 (12)	0.0439 (10)	−0.0085 (9)	0.0152 (9)	−0.0114 (9)
C31	0.0863 (16)	0.0539 (12)	0.0392 (10)	−0.0176 (11)	−0.0046 (10)	0.0067 (9)
C34	0.0725 (13)	0.0464 (11)	0.0488 (10)	−0.0088 (9)	0.0317 (10)	0.0003 (8)
C22	0.0663 (13)	0.0867 (18)	0.0395 (10)	−0.0265 (13)	0.0055 (9)	0.0027 (11)
C14	0.0606 (13)	0.109 (2)	0.0426 (11)	−0.0316 (14)	−0.0049 (10)	−0.0049 (12)
C37	0.0711 (14)	0.0378 (10)	0.0712 (14)	−0.0094 (9)	0.0088 (11)	0.0038 (10)
C13	0.0773 (16)	0.0891 (18)	0.0406 (11)	−0.0263 (14)	0.0072 (10)	−0.0181 (11)
C28	0.0535 (12)	0.0727 (16)	0.0739 (15)	0.0180 (11)	−0.0019 (11)	0.0078 (12)
C32	0.126 (2)	0.0698 (16)	0.0302 (10)	−0.0331 (16)	0.0127 (12)	0.0023 (10)
C21	0.0602 (13)	0.0862 (19)	0.0617 (14)	−0.0127 (13)	−0.0026 (11)	0.0319 (13)
C20	0.0660 (14)	0.0633 (15)	0.0866 (18)	0.0147 (12)	0.0085 (13)	0.0278 (13)
C33	0.116 (2)	0.0621 (14)	0.0454 (11)	−0.0251 (14)	0.0423 (14)	−0.0041 (10)
C30	0.093 (2)	0.087 (2)	0.0579 (15)	−0.0109 (16)	−0.0288 (15)	0.0166 (14)
C29	0.0645 (16)	0.103 (2)	0.092 (2)	0.0113 (16)	−0.0254 (15)	0.0192 (18)

### Geometric parameters (Å, °)

**Table:**

Cl1—C3	1.7219 (18)	C36—C27	1.401 (3)
Cl2—C4	1.7266 (19)	C36—C31	1.404 (3)
O1—C10	1.2141 (19)	C24—H24A	0.9700
O2—C26	1.210 (2)	C24—H24B	0.9700
C9—C10	1.529 (2)	C27—C28	1.373 (3)
C9—C8	1.541 (2)	C4—C5	1.382 (3)
C9—C17	1.560 (2)	C23—C22	1.387 (3)
C9—C25	1.603 (2)	C23—H23	0.9300
C10—C11	1.484 (2)	C5—H5	0.9300
C25—N1	1.459 (2)	C15—C14	1.376 (3)
C25—C35	1.515 (2)	C15—H15	0.9300
C25—C26	1.567 (2)	C19—C20	1.380 (3)
C17—C18	1.506 (2)	C19—H19	0.9300
C17—C24	1.516 (2)	C12—C13	1.368 (3)
C17—H17	0.9800	C12—H12	0.9300
C1—C6	1.383 (3)	C31—C30	1.409 (4)
C1—C2	1.385 (2)	C31—C32	1.412 (4)
C1—C7	1.518 (2)	C34—C33	1.413 (3)
C7—C16	1.512 (2)	C34—H34	0.9300
C7—C8	1.534 (2)	C22—C21	1.369 (4)
C7—H7	0.9800	C22—H22	0.9300
N1—C24	1.448 (2)	C14—C13	1.371 (4)
N1—C37	1.452 (2)	C14—H14	0.9300
C6—C5	1.375 (3)	C37—H37A	0.9600
C6—H6	0.9300	C37—H37B	0.9600
C8—H8A	0.9700	C37—H37C	0.9600
C8—H8B	0.9700	C13—H13	0.9300
C18—C23	1.383 (3)	C28—C29	1.410 (4)
C18—C19	1.385 (3)	C28—H28	0.9300
C26—C27	1.473 (3)	C32—C33	1.358 (4)
C2—C3	1.388 (2)	C32—H32	0.9300
C2—H2	0.9300	C21—C20	1.365 (4)
C3—C4	1.380 (3)	C21—H21	0.9300
C11—C16	1.391 (2)	C20—H20	0.9300
C11—C12	1.401 (2)	C33—H33	0.9300
C16—C15	1.396 (2)	C30—C29	1.356 (5)
C35—C34	1.367 (3)	C30—H30	0.9300
C35—C36	1.397 (3)	C29—H29	0.9300
			
C10—C9—C8	105.82 (12)	C17—C24—H24A	111.5
C10—C9—C17	111.28 (12)	N1—C24—H24B	111.5
C8—C9—C17	111.75 (12)	C17—C24—H24B	111.5
C10—C9—C25	109.40 (12)	H24A—C24—H24B	109.3
C8—C9—C25	116.04 (12)	C28—C27—C36	120.44 (19)
C17—C9—C25	102.65 (12)	C28—C27—C26	132.5 (2)
O1—C10—C11	120.92 (15)	C36—C27—C26	107.09 (16)
O1—C10—C9	122.52 (14)	C3—C4—C5	119.47 (17)
C11—C10—C9	116.55 (13)	C3—C4—Cl2	121.45 (15)
N1—C25—C35	111.22 (13)	C5—C4—Cl2	119.07 (17)
N1—C25—C26	113.12 (13)	C18—C23—C22	120.8 (2)
C35—C25—C26	101.58 (13)	C18—C23—H23	119.6
N1—C25—C9	103.72 (12)	C22—C23—H23	119.6
C35—C25—C9	115.68 (13)	C6—C5—C4	120.22 (19)
C26—C25—C9	111.90 (12)	C6—C5—H5	119.9
C18—C17—C24	116.94 (14)	C4—C5—H5	119.9
C18—C17—C9	116.44 (13)	C14—C15—C16	120.8 (2)
C24—C17—C9	103.44 (13)	C14—C15—H15	119.6
C18—C17—H17	106.4	C16—C15—H15	119.6
C24—C17—H17	106.4	C20—C19—C18	121.2 (2)
C9—C17—H17	106.4	C20—C19—H19	119.4
C6—C1—C2	118.46 (16)	C18—C19—H19	119.4
C6—C1—C7	121.07 (14)	C13—C12—C11	120.4 (2)
C2—C1—C7	120.46 (16)	C13—C12—H12	119.8
C16—C7—C1	111.05 (14)	C11—C12—H12	119.8
C16—C7—C8	112.58 (13)	C36—C31—C30	115.2 (2)
C1—C7—C8	110.86 (13)	C36—C31—C32	115.5 (2)
C16—C7—H7	107.4	C30—C31—C32	129.3 (2)
C1—C7—H7	107.4	C35—C34—C33	117.9 (2)
C8—C7—H7	107.4	C35—C34—H34	121.0
C24—N1—C37	116.63 (16)	C33—C34—H34	121.0
C24—N1—C25	108.04 (13)	C21—C22—C23	120.5 (2)
C37—N1—C25	116.51 (16)	C21—C22—H22	119.7
C5—C6—C1	121.11 (17)	C23—C22—H22	119.7
C5—C6—H6	119.4	C13—C14—C15	120.3 (2)
C1—C6—H6	119.4	C13—C14—H14	119.9
C7—C8—C9	115.71 (13)	C15—C14—H14	119.9
C7—C8—H8A	108.4	N1—C37—H37A	109.5
C9—C8—H8A	108.4	N1—C37—H37B	109.5
C7—C8—H8B	108.4	H37A—C37—H37B	109.5
C9—C8—H8B	108.4	N1—C37—H37C	109.5
H8A—C8—H8B	107.4	H37A—C37—H37C	109.5
C23—C18—C19	117.65 (18)	H37B—C37—H37C	109.5
C23—C18—C17	123.25 (17)	C12—C13—C14	120.2 (2)
C19—C18—C17	119.10 (16)	C12—C13—H13	119.9
O2—C26—C27	127.58 (16)	C14—C13—H13	119.9
O2—C26—C25	124.35 (16)	C27—C28—C29	117.0 (3)
C27—C26—C25	107.98 (14)	C27—C28—H28	121.5
C1—C2—C3	120.70 (17)	C29—C28—H28	121.5
C1—C2—H2	119.6	C33—C32—C31	120.6 (2)
C3—C2—H2	119.6	C33—C32—H32	119.7
C4—C3—C2	120.02 (16)	C31—C32—H32	119.7
C4—C3—Cl1	120.86 (14)	C20—C21—C22	119.3 (2)
C2—C3—Cl1	119.11 (15)	C20—C21—H21	120.4
C16—C11—C12	119.67 (16)	C22—C21—H21	120.4
C16—C11—C10	121.56 (15)	C21—C20—C19	120.5 (3)
C12—C11—C10	118.67 (16)	C21—C20—H20	119.7
C11—C16—C15	118.65 (17)	C19—C20—H20	119.7
C11—C16—C7	121.94 (14)	C32—C33—C34	122.9 (2)
C15—C16—C7	119.40 (16)	C32—C33—H33	118.5
C34—C35—C36	119.14 (18)	C34—C33—H33	118.5
C34—C35—C25	131.44 (18)	C29—C30—C31	121.7 (2)
C36—C35—C25	109.43 (15)	C29—C30—H30	119.1
C35—C36—C27	113.24 (16)	C31—C30—H30	119.1
C35—C36—C31	123.9 (2)	C30—C29—C28	122.7 (3)
C27—C36—C31	122.9 (2)	C30—C29—H29	118.6
N1—C24—C17	101.37 (14)	C28—C29—H29	118.6
N1—C24—H24A	111.5		

### Hydrogen-bond geometry (Å, °)

**Table:**

*D*—H···*A*	*D*—H	H···*A*	*D*···*A*	*D*—H···*A*
C8—H8A···O2	0.97	2.49	3.045 (2)	116
C17—H17···O1	0.98	2.33	2.794 (2)	108
C32—H32···O2^i^	0.93	2.48	3.351 (3)	156

Symmetry codes: (i) *x*, −*y*, *z*+1/2.
